# Water-Soluble Tomato Extract Fruitflow Alters the Phosphoproteomic Profile of Collagen-Stimulated Platelets

**DOI:** 10.3389/fphar.2021.746107

**Published:** 2021-09-27

**Authors:** Shenghao Zhang, Huilian Chen, Chuanbao Li, Beidong Chen, Huan Gong, Yanyang Zhao, Ruomei Qi

**Affiliations:** The Key Laboratory of Geriatrics, Beijing Institute of Geriatrics, Beijing Hospital, National Center of Gerontology, National Health Commission, Institute of Geriatric Medicine, Chinese Academy of Medical Sciences, Beijing, China

**Keywords:** fruitflow, platelets, phosphoproteomics, P-selectin, Akt, GSK3β

## Abstract

Platelet hyperactivity is a risk factor for cardiovascular disease and thrombosis. Recent studies reported that the tomato extract Fruitflow inhibited platelet function, but the molecular mechanism is still unclear. The present study used proteomics to quantitatively analyze the effect of fruitflow on the inhibition of collagen-stimulated platelets and validated the involvement of several signaling molecules. Fruitflow significantly inhibited human platelet aggregation and P-selectin expression that were induced by collagen. Proteomics analysis revealed that compared fruitflow-treated collagen-stimulated platelets with only collagen-stimulated platelets, 60 proteins were upregulated and 10 proteins were downregulated. Additionally, 66 phosphorylated peptides were upregulated, whereas 37 phosphorylated peptides were downregulated. Gene Ontology analysis indicated that fruitflow treatment downregulated phosphoinositide 3-kinase (PI3K)/protein kinase B and guanosine triphosphatase-mediated signal transduction in collagen-activated platelets. Biological validation indicated that fruitflow decreased Akt, glycogen synthase kinase 3β, p38 mitogen-activated protein kinase (MAPK), and heat shock protein (Hsp27) phosphorylation in collagen-stimulated platelets. Fruitflow recovered cyclic adenosine monophosphate levels in collagen-activated platelets and reduced protein kinase A substrate phosphorylation that was induced by collagen. These findings suggest that fruitflow is a functional food that can inhibit platelet function, conferring beneficial effects for people who are at risk for platelet hyperactivity-associated thrombosis.

## Introduction

Platelets are anucleate cells that play diverse roles in hemostasis and thrombosis and also contribute to immunity, inflammation, and wound healing. Platelet hyperactivity is related to hypertension, diabetes, and cardiovascular diseases (Martin et al., 2012; Thomas and Storey, 2015a; Gaiz et al., 2017). Under pathological conditions, platelets promote vascular damage and chronic inflammation by releasing various inflammatory mediators (Rendu and Brohard-Bohn, 2001; Golebiewska and Poole, 2015; Bakogiannis et al., 2019). Accumulating evidence shows that the inhibition of platelet function can decrease biological mediators of platelet secretion. The inhibition of platelet function has become an effective strategy for reducing the risk of cardiovascular disease (Duchene and von Hundelshausen, 2015; Kirichenko et al., 2016).

Studies of platelet proteomics revealed that platelets contain more than 4,000 proteins, and more than 300 protein products are released when platelets are activated (Burkhart et al., 2012). Proteomics can identify quantitative changes in the abundance and localization of thousands of proteins and various modifications, including phosphorylation, acetylation, methylation, and glycosylation (Burkhart et al., 2014; Aebersold and Mann, 2016; Manes and Nita-Lazar, 2018). The modification of phosphorylated proteins is an important biological process during platelet activation. Phosphorylated proteomics can provide useful biological information for drug target screening (Cohen, 2000; García et al., 2005; Dittrich et al., 2008). Quantitative proteomics that uses high-resolution liquid chromatography tandem mass spectrometry (LC-MS/MS) can elucidate cellular signaling cascades. Label-free proteomics utilize the signal intensity and spectral counting of peptides to quantitate both relative and absolute protein abundance, which improves the accuracy and depth of phosphoproteomics research (Megger et al., 2013; Anand et al., 2017; Manes and Nita-Lazar, 2018).

Tomatoes are one of the main vegetables in the Mediterranean diet. Tomatoes contain various nutrients that are beneficial to health. Recent studies demonstrated that the cardioprotective effects of tomato extracts are linked to the modulation of platelet function. Fruitflow is a water-soluble concentrate that mainly contains flavone, adenosine, and chlorogenic acid. O’Kennedy et al. reported that fruitflow reduced human platelet aggregation by 8–23% in an *ex vivo* preparation 3 h after administration (O'Kennedy et al., 2006; O'Kennedy et al., 2017a). Another study showed that tomato juice consumption increased erythrocyte antioxidant enzymes and decreased serum malondialdehyde in overweight and obese females (Ghavipour et al., 2015). These studies suggest that the active ingredients of tomatoes can provide health benefits. However, the inhibitory mechanism of action of fruitflow on platelet function is not fully understood.

Collagen is a powerful platelet activator that plays a critical role in thrombosis. There are three types of collagen receptors on the platelet membrane: glycoprotein Ib, glycoprotein VI, and integrin-α2β1. Collagen receptor-mediated signal transduction has been shown to be involved in platelet activation (Barnes et al., 1998; Clemetson and Clemetson, 2001; Farndale, 2006; Surin et al., 2008). To obtain further biological information about the effects of fruitflow on platelet function, we used LC-MS/MS to perform a proteomics and phosphoproteomics analysis of the effects of fruitflow in collagen-activated platelets.

## Methods

### Ethics Statement

Blood was collected from healthy donors, from whom we received written informed consent. The experiments were conducted according to the principles of the Declaration of Helsinki. The blood samples were used for the *in vitro* study. The present study was approved by the Ethics Committee of Beijing Hospital (no. 2018BJYYEC-195–02).

### Materials

Fruitflow (FF) was provided by By-Health Co., Ltd. (Zhuhai, Guangdong, China). The main biologically active ingredients of FF are adenosine, flavonoids, chlorogenic acid, phytosterols and phenolic acids, etc. (O'Kennedy et al., 2017a).

Collagen was purchased from Chrono-Log Corporation (Havertown, PA, United States). Monoclonal anti-Hsp27 and phospho-Hsp27antibodies were purchased from Abcam (Boston, MA, United States). Monoclonal anti-glycogen synthase kinase *β* (GSK3β), phospho-GSK3β, monoclonal anti- Akt, phospho-Akt, monoclonal anti-p38 MAPK, phospho-p38 MAPK, and phospho-protein kinase A (PKA) substrate (RRXS*/T*) antibodies were purchased from Cell Signaling Technology (Danvers, MA, United States). Cyclic adenosine monophosphate (cAMP) kits were obtained from R&D Systems (Minneapolis, MN, United States).

### Platelet Preparation

Venous blood was drawn from health donors who had not taken any medication in the previous 2 weeks. The blood samples were immediately mixed with 3.8% sodium citrate (1 volume of sodium citrate/9 volumes of blood) as an anticoagulant. The blood samples were then centrifuged at 500 × *g* for 15 min to obtain platelet-rich plasma. The platelet-rich plasma was diluted 1:1 with Tyrode’s/HEPES buffer (128 mM NaCl, 2.8 mM KCl, 1 mM MgCl_2_, 5 mM glucose, 12 mM NaHCO_3_, and 0.4 mM NaH_2_PO_4_, pH 7.2). To prevent platelet activation we added 2 mM ethylene glycol tetraacetic acid (EGTA) and ACD (1:10) in platelet suspension during centrifugation. The platelet suspension was centrifuged at 400 × *g* for 10 min. Platelet pellets were resuspended in Tyrode’s/HEPES buffer and centrifuged under the same conditions for 10 min. The platelet concentration was measured by ABX/HORIBA ABX Diagnostics (Montpellier, France). For Western blot, the platelet concentration was 3 × 10^9^ cells/ml.

### Measurement of Platelet Aggregation

Platelet aggregation was measured in a washed platelet suspension using a Chrono-Log aggregometer (Chrono-Log corporation, Havertown, PA, United States). Fruitflow was dissolved in 0.9% NaCl solution to prepare stock solution. The platelet suspension (1 × 10^9^) was incubated with fruitflow (100 μg/ml) for 10 min, and the cuvette was then stirred at 1,000 rotations per minute (rpm). Collagen (5 μg/ml) was added to the cuvette for 10 min at 1,000 rpm to induce platelet aggregation.

### Western Blot Analysis

The platelet suspension was incubated with 100 μg/ml fruitflow for 10 min before being stimulated with 5 μg/ml collagen for 10 min on a Chrono-Log aggregometer. Platelet lysates were analyzed by 10% sodium dodecyl sulfate-polyacrylamide gel electrophoresis and wet electrotransferred to polyvinylidene fluoride membranes. The membranes were blocked with 1% bovine serum albumin and then incubated with specific primary antibodies overnight. After three washes in PBS that contained 0.5% Tween-20, the membranes were incubated with horseradish peroxidase-conjugated secondary antibodies in TPBS for 2 h. Bands were detected by electrochemiluminescent reagent and the EvolutionCapt system (Vilber Lourmat) and quantified using Image-Pro Plus software.

### Flow Cytometry Analysis of P-Selectin Expression

The washed platelet suspension (1 × 10^6^/ml) was treated with or without fruitflow (100 μg/ml) for 10 min. Afterward, collagen (5 μg/ml) was added for another 10 min at 37°C. The platelets were fixed by the addition of 4% paraformaldehyde for 10 min. After washing three times, the platelets were incubated with PE**-**conjugated CD62P and FITC-conjugated CD61 for 30 min. The platelets were analyzed on a FACScan flow cytometer (BD Bioscience) with 10,000 events per gate and analyzed using FlowJo software.

### Preparation and Digestion of Proteins

Washed platelets were used in the present study. The platelet suspension (1 × 10^9^cell/ml) was incubated with 100 μg/ml fruitflow for 10 min, and then 5 μg/ml collagen was added for 10 min. The samples were centrifugated at 16,000 × *g* for 3 min at 4°C and resuspended in 500 μL cold phosphate-buffered saline (PBS), repeated twice, and then 500 μl UA lysis buffer (8 M Urea and 150 mM Tris-HCl, pH 8.0) was added to the samples, followed by storage at −80 °C.

### Phosphopeptides Enrichment and LC-MS/MS Analysis

Label-free proteomics analysis was performed by Applied Protein Technology (Shanghai, China). LC-MS/MS spectra were searched using a Q Exactive HF/HFX mass spectrometer coupled to Easy nLC (Thermo Fisher Scientific), which is controlled by IntelliFlow technology. Immobilized metal affinity chromatography (IMAC) was used to enrich phosphopeptides. According to the manufacturer’s instructions (Thermo Scientific), the enrichment was carried out using High-SelectTM Fe-NTA Phosphopeptides Enrichment Kit. The MS raw data for each sample were combined and searched using MaxQuant 1.5.3.17 software for the identification and quantification analysis. A false discovery rate <1% was applied. Proteomic samples were analyzed by LC-MS/MS as described in Supplement S1. Gene Ontology analysis was performed at https://david.ncifcrf.gov/home.jsp.

### Measurement of cAMP by Enzyme-Linked Immunosorbent Assay

Washed platelets were treated with or without fruitflow (100 μg/ml) for 10 min, and then collagen (5 μg/ml) was added for 10 min. After centrifugation at 9,600 *g* for 10 min, the supernatant was collected to measure cAMP using enzyme-linked immunosorbent assay (ELISA) kits according to the manufacturer’s instructions.

### Statistical Analysis

Quantitative data are presented as the mean ± SEM. Significant differences between two groups were analyzed using two-tail paired Student’s *t*-test. All of the analyses were performed using Prism 8.3 software (GraphPad, San Diego, CA, United States). Values of *p* < 0.05 were considered statistically significant.

## Results

### Fruitflow Inhibited Platelet Aggregation and P-Selectin Expression in Collagen-Stimulated Platelets

**FIGURE 1:**
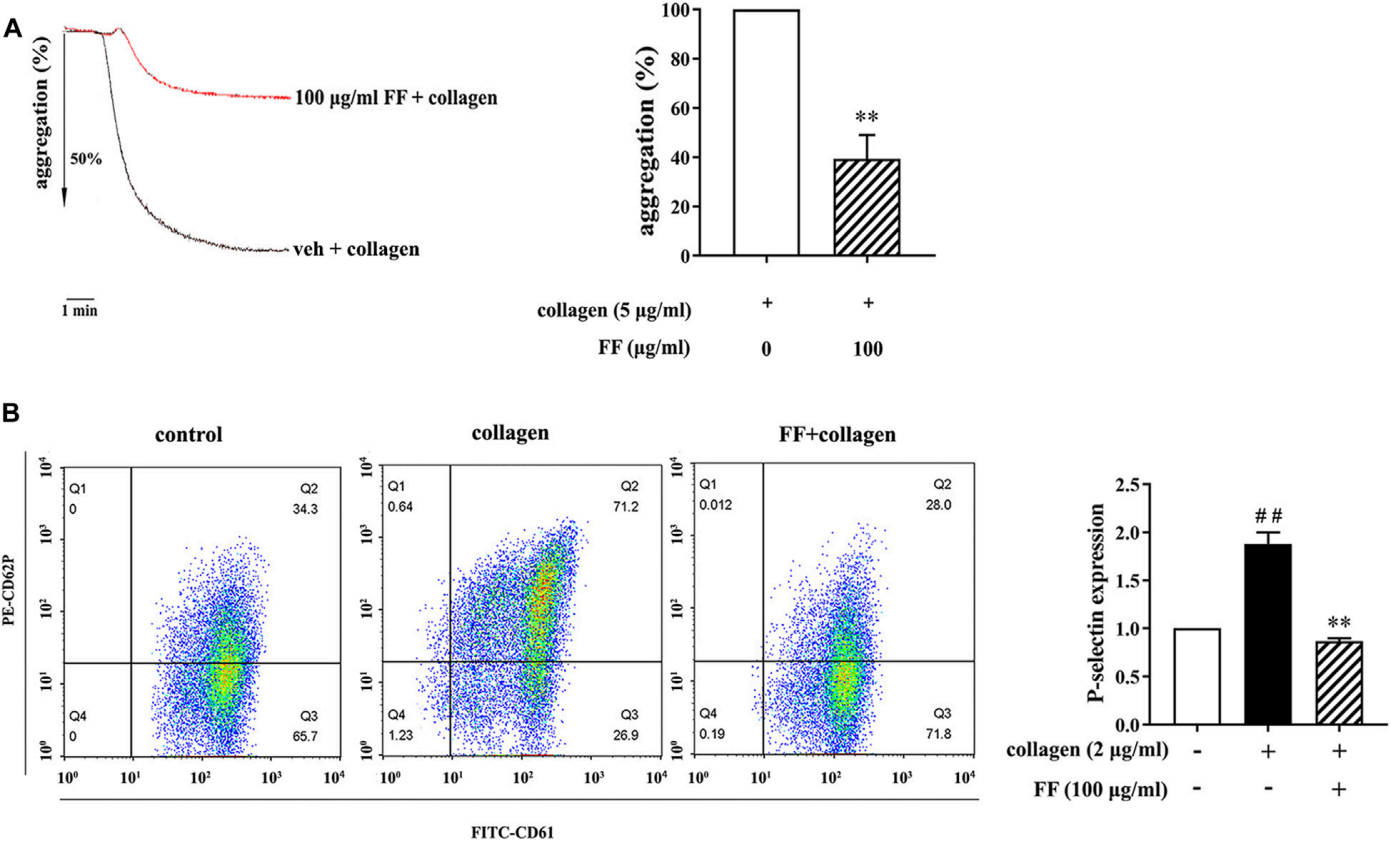
Fruitflow inhibited platelet aggregation and P-selectin expression in collagen-stimulated platelets. Platelets were treated with vehicle (0.9% NaCl) or FF (100 μg/ml) for 10 min, and then collagen was added for another 10 min. **(A)** Fruitflow inhibited platelet aggregation that was induced by collagen. Ordinate value is aggregation ratio and abscissa value is time (min). **(B)** Flow cytometry analysis of P-selectin expression. Fruitflow inhibited P-selectin expression that was induced by collagen. The data represent three independent experiments. *p < 0.05, **p < 0.01, significant difference between FF-treated collagen-activated platelets and collagen-activated platelets alone. ^#^ *p* < 0.05, significant difference between collagen-treated platelets and control.

Platelet aggregation and P-selectin expression are important biological processes in platelet activation. We first determined the effect of fruitflow on platelet aggregation and P-selectin expression in collagen-activated platelets. Based on our pre-experiments, in which fruitflow (1, 10, 30, and 100 μg/ml) dose-dependently inhibited collagen-induced platelet aggregation, we used 100 μg/ml fruitflow in the present study. As shown in Figure 1, 100 μg/ml fruitflow significantly inhibited platelet aggregation, in which the aggregation ratio decreased by 60.7 ± 9.7%. We also analyzed the effect of fruitflow on P-selectin using flow cytometry. Collagen increased P-selectin expression by 87.9% on the platelet membrane, whereas 100 μg/ml fruitflow completely suppressed P-selectin expression that was induced by collagen.

### Proteomics Analysis of Fruitflow Treatment and No Treatment in Collagen-Activated Platelets

**FIGURE 2:**
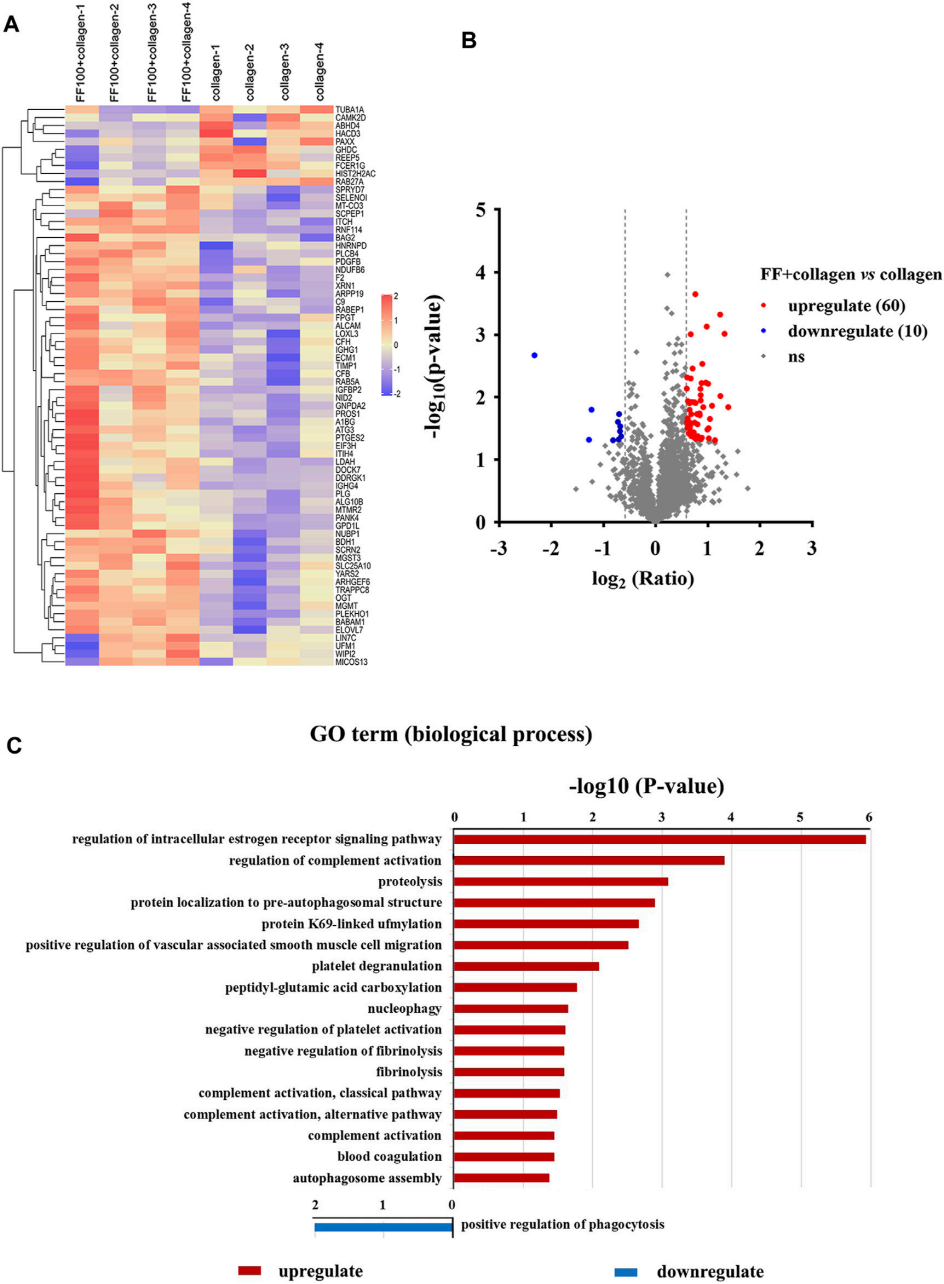
Identification of proteins with and without FF treatment in collagen-stimulated platelets. Washed human platelets were incubated with FF (100 μg/ml) for 10 min, and then collagen (5 μg/ml) was added for 10 min. **(A)** Heatmap of the identification and quantification of proteins in FF-treated collagen-stimulated platelets and collagen-stimulated platelets alone. **(B)** Volcano map of the identification and quantification of proteins in FF-treated collagen-stimulated platelets and collagen-stimulated platelets alone. Red, blue, and gray clusters indicate up-, down-, and unregulated proteins, respectively. **(C)** Biological processes identified by Gene Ontology enrichment analysis of collagen-stimulated platelets with and without FF treatment.

To explore the mechanism of action fruitflow on platelet function, we performed proteomics analysis of fruitflow treatment and no treatment in collagen-activated platelets. Platelets were pretreated with fruitflow for 10 min, and collagen was then added for 10 min. The proteomics analysis identified 3,856 proteins, and 3,182 proteins were quantified. Different proteomic profiles were found between fruitflow treatment and no treatment in collagen-stimulated platelets. As shown in Figure 2, compared fruitflow-treated collagen-stimulated platelets with only collagen-stimulated platelets, 60 proteins were upregulated and 10 proteins were downregulated (*p* < 0.05). The Gene Ontology analysis showed different biological processes that were associated with fruitflow treatment and no treatment in collagen-stimulated platelets. Upregulated biological processes included platelet degranulation, nucleophage/autophagosome assembly, fibrilysis, blood coagulation, negative regulation of platelet activation, and negative regulation of fibrinolysis, etc. Detailed data was shown in Supplement S2 Table 1.

### Phosphoproteomics Analysis of Fruitflow Treatment and No Treatment in Collagen-Activated Platelets

**FIGURE 3:**
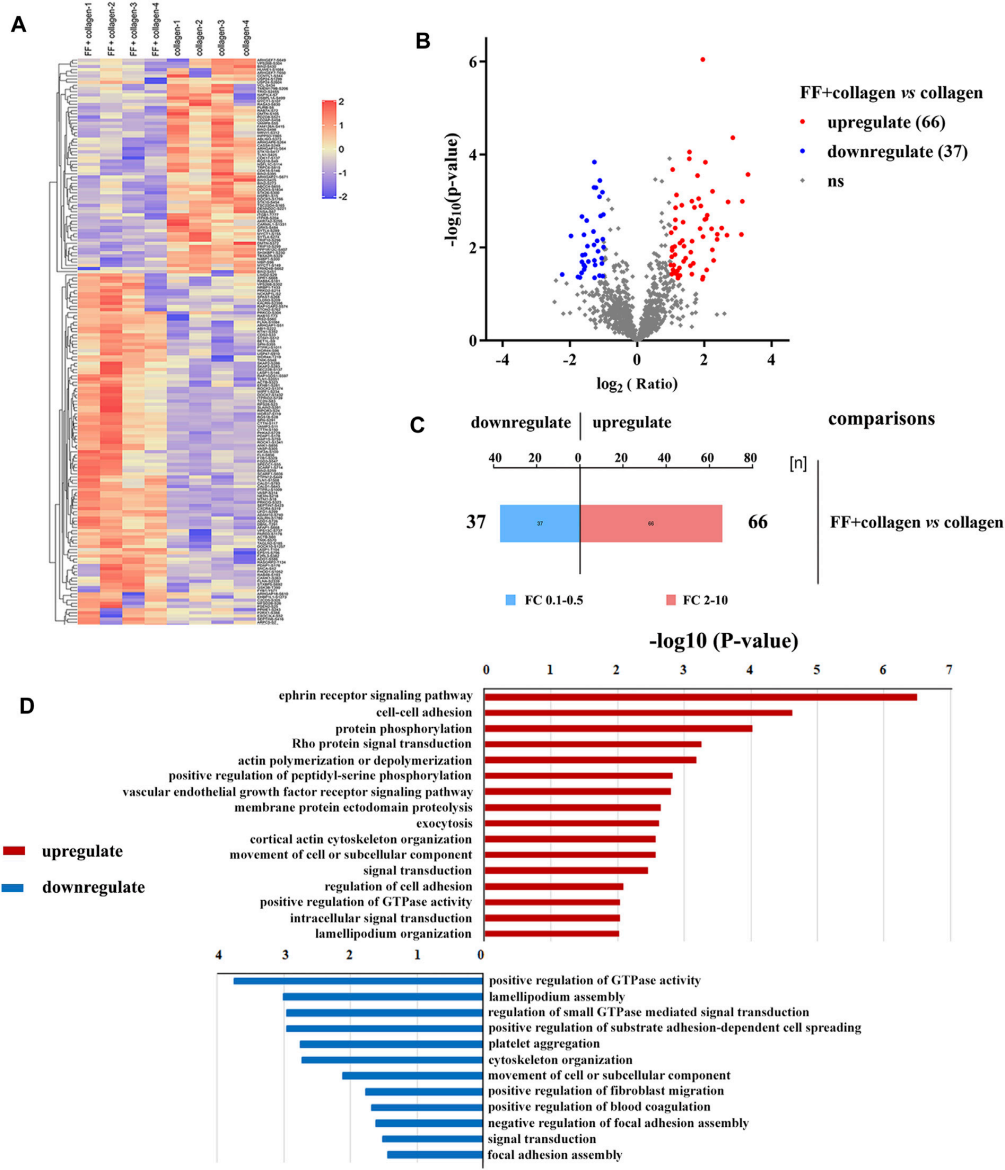
Quantitative phosphoproteomics analysis of collagen-stimulated platelets with and without FF treatment. **(A)** Heatmap of different protein phosphorylation profiles with and without FF treatment in collagen-stimulated platelets. **(B)** Volcano map that shows 66 upregulated and 37 downregulated phosphopeptides with and without FF treatment, respectively, in collagen-stimulated platelets. **(C)** Histogram of multiple correlations of collagen-stimulated platelets with and without FF treatment. **(D)** Biological processes identified by Gene Ontology enrichment analysis of collagen-stimulated platelets with and without FF treatment.

To explore the effect of fruitflow on collagen-stimulated platelets, we conducted a quantitative phosphosproteomics analysis. The quantitative analysis detected 2,099 phosphorylated peptides and 2,376 phosphorylated sites in 1,051 phosphorylated proteins. As shown in Figure 3, a significant difference was found between fruitflow treatment and no treatment in collagen-activated platelets. Compared FF-treated collagen-stimulated platelets with only collagen-stimulated platelets, 66 phosphorylated peptides were upregulated two time, whereas 37 phosphorylated peptides were downregulated 0.5 times. As shown in Table 1, vasodilator-stimulated phosphoprotein (VASP) phosphorylation levels were upregulated in FF-treated platelets. VASP is a substrate of protein kinase G activation, and it interacts with nitric oxide through the soluble guanylate cyclase (sGC)/cyclic guanosine monophosphate (cGMP) pathway (Li et al., 2003). Most of the phosphorylated proteins that increased are related to calcium mobilization (calcium/calmodulin-dependent protein kinase, CAMK1, tyrosine phosphatase (PTPRJ, PTPN12) and actin polymerization (Rho-associated protein kinase, ROCK). The phosphorylated proteins that decreased included serine/threonine kinase (STK10), thromboxane receptor (TBXA2R**)**, and heat shock 27 kDa protein 1 (HSPB1, Hsp27). Interestingly, INPP5D (namely SH2 domain-containing inositol-5′-phosphatase 1, SHIP1) and INPPL1 (namely SH2 domain-containing inositol-5′-phosphatase 2, SHIP2) only existed in fruitflow-treated collagen-stimulated platelets, but not in collagen-stimulated platelets (in Supplement 4 Table 3). The function of INPPL1 is to specifically hydrolyze the 5-phosphate of phosphatidylinositol-3,4,5-trisphosphate (PtdIns [3,4,5]P_3_) to produce PtdIns(3,4)P_2_, thereby negatively regulating the phosphoinositide-3 kinase (PI3K) pathway (Backers et al., 2003; Durrant et al., 2017; Fu et al., 2019). This suggests that inhibition of the PI3K/protein kinase B (Akt) pathway might be an important mechanism by which fruitflow suppresses platelet function. Detailed phosphoproteomic data was shown in Supplement 3 Table 2 and Supplement 4 Table 3.

**TABLE 1:** Different phosphorylated proteins in FF + collagen-*vs* collagen-treated platelets.

Uniprot	Gene	Site	Regulation
Q13464	ROCK1	Ser1341	up
O75116	ROCK2	Ser1374	up
Q7LDG7	RASGRP2	Ser116	up
P50552	VASP	Ser305/Ser314	up
Q05209	PTPN12	Ser449	up
O15117	FYB1	Ser329	up
O75563	SKAP2	Ser286/Ser283	up
Q684P5	RAP1GAP2	Ser574	up
P49841	GSK3B	Thr390	up
Q12913	PTPRJ	Ser1374	up
Q04759	PRKCQ	Ser1011	up
Q07960	ARHGAP1	Ser51	up
Q14012	CAMK1	Ser363	up
Q13496	MTM1	Ser18	up
Q9BZL6	PRKD2	Ser214	up
P21731	TBXA2R	Ser329	down
P34947	GRK5	Ser484	down
Q14155	ARHGEF7	Ser650	down
Q96B97	SH3KBP1	Ser230	down
O94804	STK10	Ser454/Ser417	down
P04792	HSPB1	Ser15	down
O43182	ARHGAP6	Ser105	down
Q14644	RASA3	Ser830	down

### Validation of the Effect of Fruitflow in Collagen-Stimulated Platelets

**FIGURE 4:**
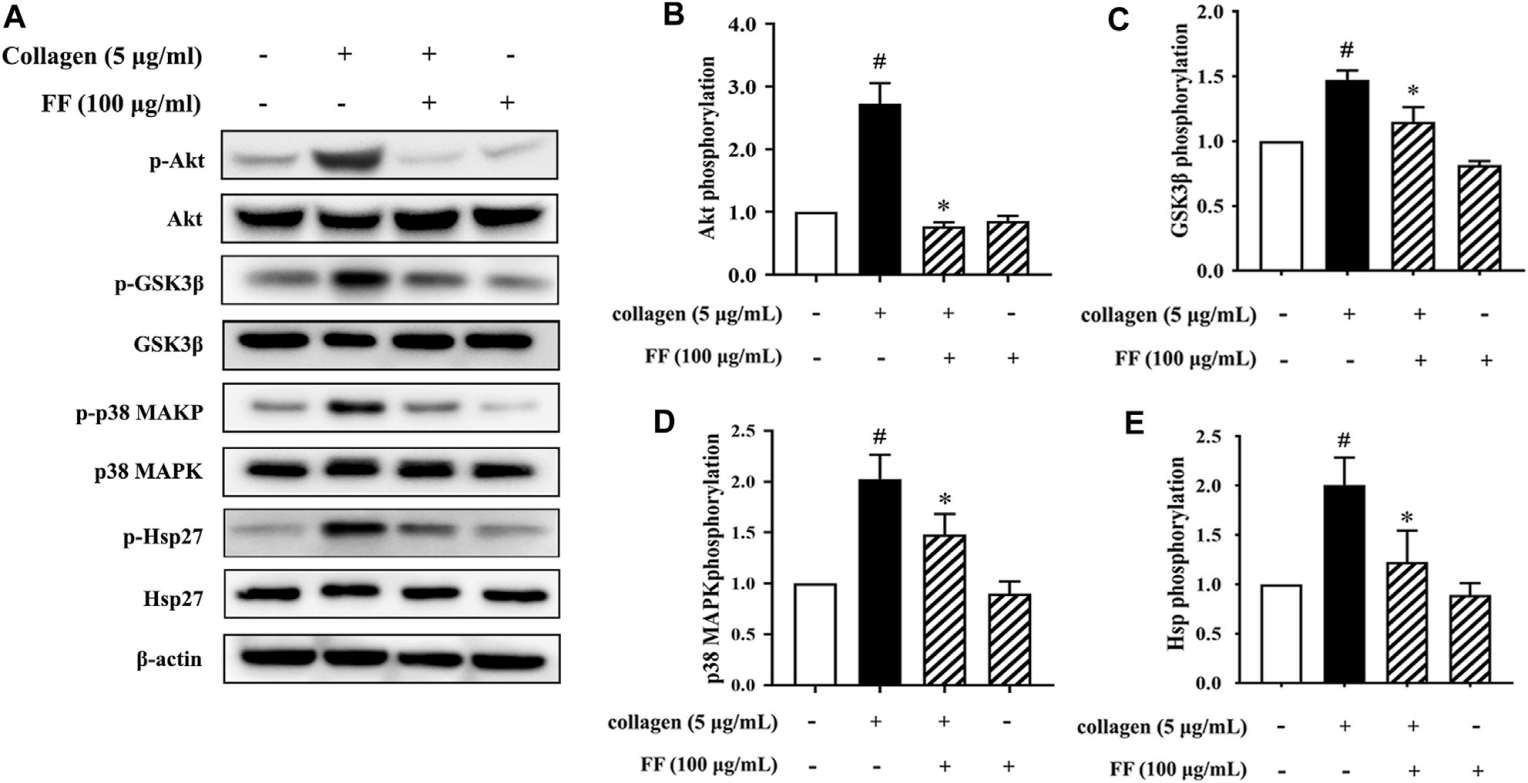
Validation of effect of FF on Akt, GSK3β, p38 MAPK, and Hsp phosphorylation in collagen-stimulated platelets. **(A)** Fruitflow suppressed Akt, GSK3β, p38 MAPK, and Hsp phosphorylation in collagen-stimulated platelets. **(B)** Density analysis of Akt phosphorylation in FF-treated collagen-stimulated platelets and collagen-stimulated platelets alone. **(C)** Density analysis of GSK3β phosphorylation in FF-treated collagen-stimulated platelets and collagen-stimulated platelets alone. **(D)** Density analysis of p38 MAPK phosphorylation in FF-treated collagen-stimulated platelets and collagen-stimulated platelets alone. **(E)** Density analysis of Hsp phosphorylation in FF-treated collagen-stimulated platelets and collagen-stimulated platelets alone. The data represent three independent experiments. *p < 0.05, significant difference between FF-treated collagen-stimulated platelets and collagen-stimulated platelets alone. ^#^ *p* < 0.05, significant difference between collagen-treated platelets and control.

Based on the research background of collagen-mediated signaling pathway upon platelet activation and the phosphoproteomics data, we verified some important molecular. The phosphoproteomics showed that several phosphorylated peptides were linked to PI3K/Akt pathway. Therefore, we determined the effect of fruitflow on the phosphorylation of Akt and its downstream molecular GSK3β. As shown in Figure 4, 100 μg/ml fruitflow pretreatment completely suppressed Akt and GSK3β phosphorylation that was induced by collagen.

Moreover, there were four phosphorylated peptides of the MAPK families presented in Supplement 3 Table 2. They were MAPKAPK2, MAPK14, MAPKAP1 and MAP4K2. We validated the phosphorylation of p38 MAPK and its downstream molecular HSPB1(Hsp27).

The same dose of fruitflow effectively inhibited p38 MAPK and Hsp27 phosphorylation that was induced by collagen.

### Fruitflow Recovered cAMP Levels and Inhibited the Phosphorylation of PKA Substrates in Collagen-Stimulated Platelets

**FIGURE 5:**
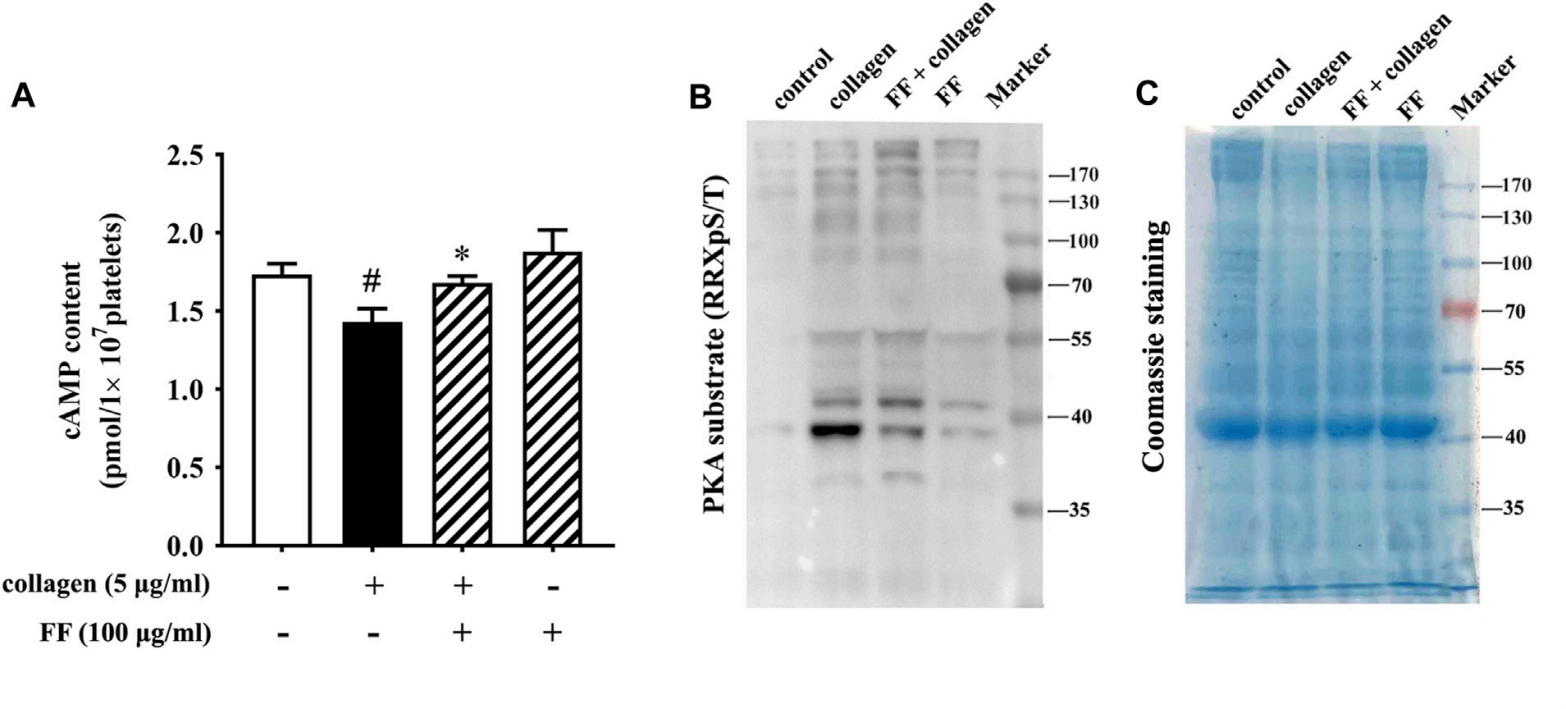
Fruitflow recovered cAMP levels and reduced the phosphorylation of PKA substrates in collagen-stimulated platelets. **(A)** Analysis of cAMP levels by ELISA. **(B)** Western blot analysis of the phosphorylation of PKA substrates. **(C)** Coomassie staining showed that protein abundance was equal. The data represent three independent experiments. *p < 0.05, significant difference between FF-treated collagen-stimulated platelets and collagen-stimulated platelets alone. ^#^ *p* < 0.05, significant difference between collagen-treated platelets and control.

The phosphoproteomics revealed that several substrates of PKA were significantly different between the FF-treated collagen-stimulated platelets and the collagen-stimulated platelets alone. They were VASP (vasodilation stimulating protein), FLNA (filament protein A), HSP27 (heat shock protein 27) and Rap1GAP2 (activator protein of GTPase-Rap1b) (in Supplement 3 Table 2). Protein kinase A (PKA) is a downstream molecule regulated by cyclic adenosine monophosphate (cAMP). cAMP is a second messenger that plays a negative regulatory role in platelet activation (Fuentes and Palomo, 2014; Raslan and Naseem, 2014). We investigated whether fruitflow can affect cAMP/PKA pathway. As shown in Figure 5, collagen stimulation decreased cAMP levels, and fruitflow treatment restored cAMP levels that were decreased by collagen. Collagen stimulation increased the phosphorylation of PKA substrates, and 100 μg/ml fruitflow abolished the phosphorylation of PKA substrates.

## Discussion

Several recent studies reported that the water-soluble tomato extract fruitflow inhibits platelet aggregation (Uddin et al., 2018; O'Kennedy et al., 2017b). In the present study, we confirmed that fruitflow inhibited platelet aggregation and P-selectin expression in collagen-activated platelets. P-selectin is an important marker of platelet activation that acts as a bridging molecule to recruit inflammatory cells to adhere to endothelial cells (Thomas and Storey, 2015b; Kappelmayer and Nagy, 2017). Compared with resting platelets, collagen-stimulated platelets exhibited a decrease in cAMP levels and an increase in the phosphorylation of PKA substrates, and fruitflow reversed these changes. However, the mechanism by which cAMP/PKA signaling regulates platelet function remains unclear.

Interestingly, our results revealed a significant difference in phosphoproteomic profiles between fruitflow treatment and no treatment in collagen-stimulated platelets. The upregulated biological processes included cell-cell adhesion, protein phosphorylation, Rho protein signal transduction, and actin polymerization. The downregulated biological processes included positive regulation of GTPase activity, positive regulation of substrate adhesion-dependent cell spreading, and platelet aggregation. The phosphoproteomics analysis also revealed protein modification-related biological processes, cell composition changes, and more protein phosphorylated sites. We verified several GTPase signal transduction and PI3K/Akt pathway-related kinase signaling molecules by Western blot. Collagen stimulation increased Akt, GSK3β, p38 MAPK, and Hsp27 phosphorylation, and fruitflow treatment significantly inhibited their phosphorylation. Previous studies showed that Akt, GSK3β, p38 MAPK, and Hsp27 are involved in collagen- and thrombin-induced platelet activation (O'Brien et al., 2012; Moore et al., 2013; Liu et al., 2018; Saklatvala et al., 1996). These findings were consistent with the phosphorylated proteomics analysis. The Gene Ontology analysis revealed that fruitflow treatment downregulated GTPase-mediated signal transduction. This indicates that fruitflow inhibits platelet function through multiple targets. The quantitative phosphoproteomics analysis by MS further provided important biological information to understand the mechanism by which fruitflow inhibits platelet activation. However, the mechanism of action of fruitflow on interactions between signaling molecules needs further investigation.

Tomatoes are the most popular vegetable worldwide, especially in Mediterranean countries. Tomatoes contain various biologically active ingredients, among which lycopene has been shown to exert a protective effect on the enlarged prostate (Campbell et al., 2004; Mordente et al., 2011). Previous studies showed that daily 65 mg fruitflow administration partly suppressed platelet function (O'Kennedy et al., 2017b). Our previous clinic trial showed that daily 150 mg fruitflow intervention for 7 days could reduce ADP and collagen-induced platelet aggregation by 7.7 and 10.2% in elderly subjects. Fruitflow was the first product in Europe to obtain an approved, proprietary health claim under Article 13 (5) of the European Health Claims Regulation 1924/2006 on nutrition and health claim made on foods (19). A previous study showed that an aqueous extract of tomato dose-dependently inhibited plasma anti-antitensin converting enzyme factor (Biswas et al., 2014). Our data provide some novel evidence of the inhibition of platelet function by fruitflow.

The limitation of the present study was that we have only verified the phosphorylation changes of a few molecules, but the interaction between these proteins is still unclear. The results of proteomics also suggest that fruitflow may affect calcium mobilization, and this mechanism needs to be further explored.

**FIGURE 6:**
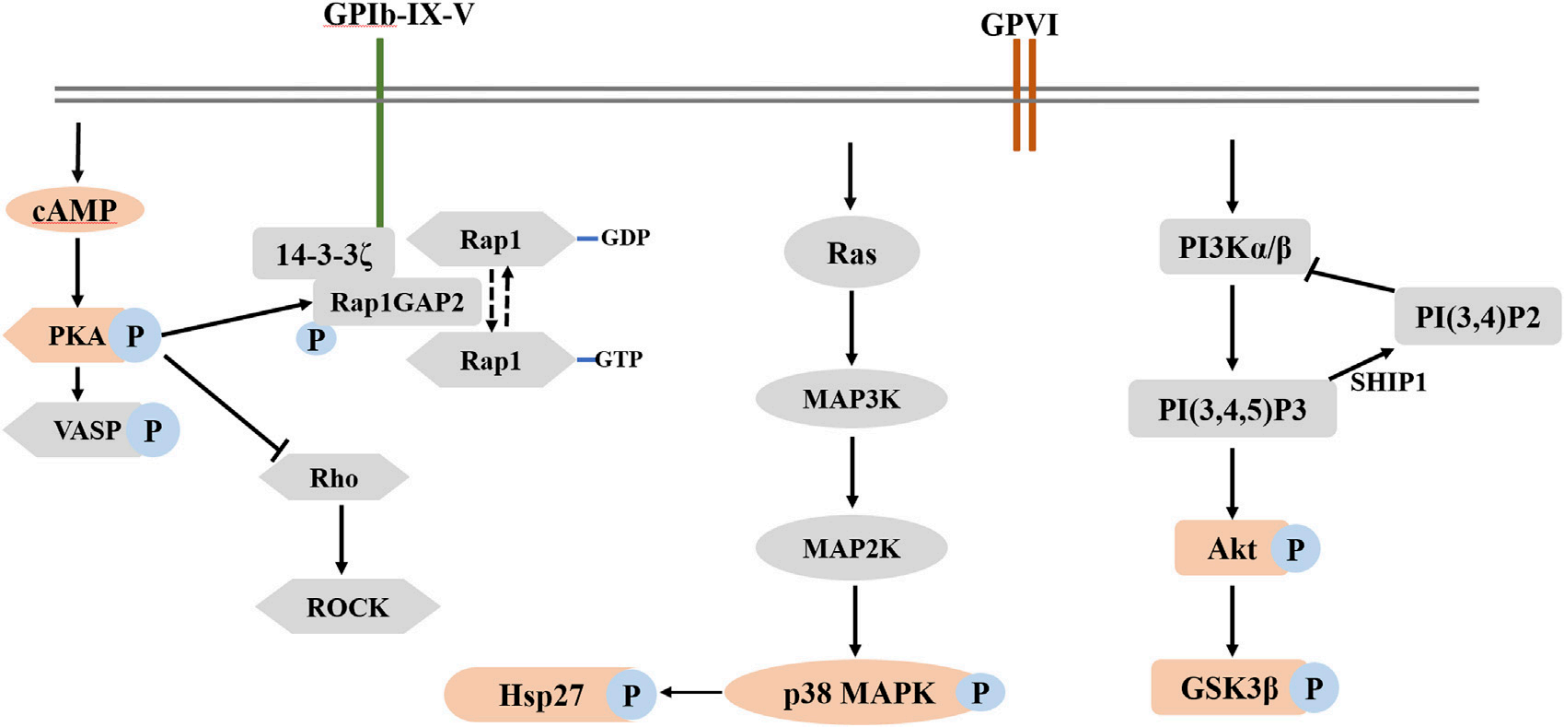
Potential mechanism of action of fruitflow in collagen-stimulated platelets.

In conclusion, the present study showed that fruitflow inhibited platelet aggregation and P-selectin expression in collagen-stimulated human platelets. We first applied proteomics and phosphoproteomics approaches to comprehensively investigate the effect of fruitflow on collagen-activated platelets. Proteomics analysis revealed that compared fruitflow-treated collagen-stimulated platelets with only collagen-stimulated platelets, 60 proteins were upregulated and 10 proteins were downregulated. Additionally, 66 phosphorylated peptides were upregulated, whereas 37 phosphorylated peptides were downregulated. Biological verification indicated that the mechanism of action of fruitflow in inhibiting platelet function is related to the suppression of Akt, GSK3β, p38 MAPK, and Hsp27 phosphorylation in collagen-stimulated platelets. Overall, fruitflow can inhibit platelet function and modify proteins in collagen-stimulated platelets, suggesting that fruitflow can provide health benefits for people who are at risk of platelet hyperactivity-related thrombosis though inhibiting platelet function. Potential mechanism of action of fruitflow in collagen-stimulated platelets was shown in Figure 6.

## Data Availability

The data presented in the study are deposited to the ProteomeXchange Consortium (http://proteomecentral.proteomexchange.org) via the iProX partner repository, accession number PXD027834.
