# Supplementary material for: Water-Soluble Tomato Extract Fruitflow Alters the Phosphoproteomic Profile of Collagen-Stimulated Platelets
Source: Front Pharmacol. 2021 Sep 27;12:746107. doi: 10.3389/fphar.2021.746107 (PMC8502824; doi:10.3389/fphar.2021.746107)
Supplement: Supplementary file 3 [file Presentation1.pdf]

Supplement 1 method

Label-free phosphoproteomics analysis

All steps of the analytical workflow, including sample preparation, proteolytic digestion, quality control, global proteome analysis, phosphopeptid enrichment, mass spectrometry, spectrum processing, database searching and quantification. A concentration of 3 × 10^8 platelets/ml were centrifugated at 16,000 × g for 3 minutes at 4°C and resuspended in 500 µl cold PBS. Repeat the above operation twice. Samples were stopped by adding UA lysis buffer (8 M Urea, 150 mM Tris-HCl pH 8.0). Lysed samples were immediately shock-frozen in liquid nitrogen and store at -80°C. 200 µg of proteins for each sample were processed separately and digested with trypsin. Digest efficiency and reproducibility were quality controlled prior to desalted on C18 Cartridges (Empore™ SPE Cartridges C18 (standard density), bed I.D. 7 mm, volume 3 ml, Sigma), concentrated by vacuum centrifugation and reconstituted in 40 µl of 0.1% (v/v) formic acid. The peptide content was estimated by UV light spectral density at 280 nm using an extinctions coefficient of 1.1 of 0.1% (g/l) solution. After global proteome quantification, immobilized metal affinity chromatography (IMAC) was used to enrich phosphopeptides. According to the manufacturer's instructions (Thermo Scientific), the enrichment was carried out using High-SelectTM Fe-NTA Phosphopeptides Enrichment Kit, which enables fast and efficient enrichment of phosphorylated peptides with greater than 90% specificity. Each spin column included in the kit contains a phosphopeptide specific resin that offers excellent binding and recovery properties for enriching up to 150 µg of phosphopeptides per column.

LC-MS/MS analysis was performed on Q Exactive HF/HFX mass spectrometer coupled to Easy nLC (Thermo Fisher Scientific) controlled by IntelliFlow technology. A false discovery rate of < 1% was applied and phosphorylation site localization were determined by the phosphoRS algorithm. The MS raw data for each sample were combined and searched using the MaxQuant (1.5.3.17) software for identification and quantitation analysis.)
